# Can the hummingbird sign contribute to the diagnosis of idiopathic normal pressure hydrocephalus?

**DOI:** 10.55730/1300-0144.5505

**Published:** 2022-07-23

**Authors:** Halil ÖNDER, Selçuk ÇOMOĞLU

**Affiliations:** Department of Neurology, Dışkapı Yıldırım Beyazıt Training and Research Hospital, Ankara, Turkey

**To the Editor**,

We read with great interest the article by Atalay et al. in which they illustrate the frequent presence of hummingbird signs in their patient group with idiopathic normal pressure hydrocephalus (iNPH) [1]. The results are substantially interesting; however, we believe that some points may be further deliberated for a better understanding of this crucial study.

The authors found the presence of the hummingbird sign in 92.3% of their iNPH subjects. Remarkably, a perfect agreement for the hummingbird signs was met between three observers [1], increasing the reliability of the data. Besides, they found a statistically significant correlation between the presence of hummingbird signs and vascular compression findings in the patient group, whereas no correlation was found between these parameters in the control group [1]. In conclusion of these findings, firstly, they suggested to use the hummingbird sign as a radiological supportive finding for iNPH [1]. Secondly, they hypothesized that tortuous posterior circulation may cause upper displacement of the floor of the third ventricle and mimic hummingbird signs in iNPH patients. The hummingbird sign refers to the neuroimaging appearance of the disproportional atrophy of the midbrain in comparison to the rather preserved pons which is known as a neuroimaging sign of PSP with high diagnostic accuracy [2]. Considering that PSP is a neurodegenerative disease and the reduction in the brainstem volume, we observe reflects atrophy of this region, hypothesizing a view of physical compression of the brainstem as an explanation may be highly speculative. To support such a hypothesis, the demonstration of the reversibility of this appearance is required. As also referred by the authors [1], the literature, in this regard, consists of a unique study on 20 iNPH subjects showing increase of midbrain diameters after shunting [3] and a unique case report of iNPH where the hummingbird sign disappears after shunt surgery [4]. However, in the study reporting changes in the midbrain diameter measurements after shunt surgery, patients met the diagnosis of probable iNPH [3]. Besides, data regarding the long-term follow-up after surgery were not included in that case report, preventing to exclude an underlying neurodegenerative pathology [4]. On the other hand, in a study by Hiraoka et al. [5], which was also referred by Atalay et al. [1], no significant difference in midbrain measurements were detected after the shunt operation [5]. It is remarkable to state that all patients included in this study [5] were those who had shown clinical improvements after shunt surgery, meeting the definite criteria for iNPH [5, 6]. In light of these data, we also think that the selection method of the patients in the study by Atalay et al. [1] may be further interrogated. Notably, the authors formed the patient group from those with a diagnosis of possible iNPH, and no data regarding the response to cerebrospinal fluid (CSF) diversion was noted. In clinical practice, the differential diagnosis of iNPH constitutes strictly a challenging issue. The gold standard of the diagnosis is the short-term response to CSF drainage [7] and the clinical improvement after surgery is required for the definite diagnosis of iNPH [6]. Besides, many pitfalls associated with the diagnosis of iNPH still exist and, among them, the comorbidity of a possible neurodegenerative disorder in iNPH has gained attention in many recent reports [7, 8]. Such that, Espay et al. reported that postshunt benefits in patients with initial diagnosis of iNPH persisted in only 32% of patients at 36 months, with a known revised diagnosis of a neurodegenerative disease in over 25% [7]. At this point, a particular focus has been given to the clinical and pathological studies showing the cooccurrence of progressive supranuclear palsy (PSP) pathology in patients with initial diagnosis of iNPH [7, 8]. A crucial issue discussed in the related recent reports is the overlap in the neuroimaging findings between PSP and iNPH subjects [9, 10]. We had also discussed previously that an underlying NPH pathophysiology might not be excluded in those patients with PSP showing iNPH-like MRI features [10].

Taken together, we think that the high prevalence of the hummingbird sign in the study of Atalay et al. may be associated with possible underlying comorbid PSP pathology in some of those patients which could not be excluded via the patient selection method. To clarify this issue, future prospective studies including a large group of patients with a definitive iNPH diagnosis are warranted. The results of these studies may provide crucial contributions regarding the diagnostic process of iNPH. Besides, these study results may enlighten the pathophysiological role of the brainstem in iNPH symptomatology, and also its role in the clinical manifestations of neurodegenerative diseases such as PSP.
